# 2-Hy­droxy-2,3,5,10,11,11a-hexa­hydro-1*H*-pyrrolo­[2,1-*c*][1,4]benzodiazepine-5,11-dione

**DOI:** 10.1107/S1600536811025244

**Published:** 2011-07-06

**Authors:** Sarah Ourahou, Hafid Zouihri, Mohamed Massoui, El Mokhtar Essassi, Seik Weng Ng

**Affiliations:** a Laboratoire de Chimie Organique Hétérocyclique, Pôle de Compétences Pharmacochimie, Université Mohammed V-Agdal, BP 1014 Avenue Ibn Batout, Rabat, Morocco; b CNRST, Division of UATRS, Angle Allal Fassi/FAR, BP 8027, 10000 Rabat, Morocco; c Department of Chemistry, University of Malaya, 50603 Kuala Lumpur, Malaysia; d Chemistry Department, Faculty of Science, King Abdulaziz University, PO Box 80203 Jeddah, Saudi Arabia

## Abstract

The seven-membered ring of the title compound, C_12_H_12_N_2_O_3_, which is fused with the phenyl­ene ring, adopts a boat-shaped conformation (with the methine C atom as the prow and the phenyl­ene C atoms as the stern); the H atom on the methine linkage exists in an axial position. The five-membered ring that is fused with the seven-membered ring adopts an envelope conformation (with the C atom bearing the hy­droxy substituent representing the flap) [the deviation from the plane defined by the other four atoms is 0.200 (7) Å in one mol­ecule and 0.627 (5) Å in the other]. The two independent mol­ecules are disposed about a pseudo center of inversion and are connected by a pair of N—H⋯O hydrogen bonds. Adjacent dimers are linked by a pair of O—H⋯O hydrogen bonds, generating a chain running along the *b* axis.

## Related literature

For the structure of *cyclo*-(anthranoyl-prol­yl), the compound without the hy­droxy substituent, see: Feigel *et al.* (1990 ▶).
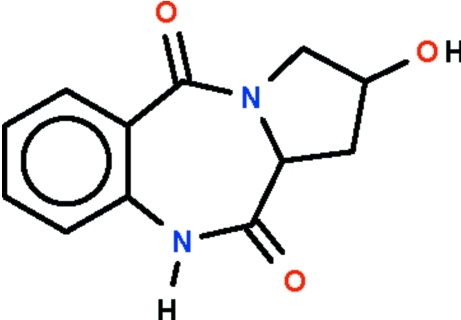


## Experimental

### 

#### Crystal data

C_12_H_12_N_2_O_3_
*M* *_r_* = 232.24
Monoclinic, 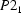

*a* = 4.8366 (2) Å
*b* = 25.7449 (11) Å
*c* = 8.5420 (4) Å
β = 96.509 (2)°
*V* = 1056.77 (8) Å^3^
*Z* = 4
Mo *K*α radiation
μ = 0.11 mm^−1^
*T* = 293 K
0.20 × 0.15 × 0.10 mm

#### Data collection

Bruker APEXII CCD-detector diffractometer
7493 measured reflections
2435 independent reflections
2027 reflections with *I* > 2σ(*I*)
*R*_int_ = 0.032

#### Refinement

*R*[*F* ^2^ > 2σ(*F* ^2^)] = 0.044
*wR*(*F* ^2^) = 0.110
*S* = 1.03
2435 reflections
309 parameters
1 restraint
H-atom parameters constrained
Δρ_max_ = 0.40 e Å^−3^
Δρ_min_ = −0.34 e Å^−3^

### 

Data collection: *APEX2* (Bruker, 2005 ▶); cell refinement: *SAINT* (Bruker, 2005 ▶); data reduction: *SAINT*; program(s) used to solve structure: *SHELXS97* (Sheldrick, 2008 ▶); program(s) used to refine structure: *SHELXL97* (Sheldrick, 2008 ▶); molecular graphics: *X-SEED* (Barbour, 2001 ▶); software used to prepare material for publication: *publCIF* (Westrip, 2010 ▶).

## Supplementary Material

Crystal structure: contains datablock(s) global, I. DOI: 10.1107/S1600536811025244/zs2124sup1.cif

Structure factors: contains datablock(s) I. DOI: 10.1107/S1600536811025244/zs2124Isup2.hkl

Supplementary material file. DOI: 10.1107/S1600536811025244/zs2124Isup3.cml

Additional supplementary materials:  crystallographic information; 3D view; checkCIF report

## Figures and Tables

**Table 1:** Hydrogen-bond geometry (Å, °)

*D*—H⋯*A*	*D*—H	H⋯*A*	*D*⋯*A*	*D*—H⋯*A*
O3—H3o⋯O5^i^	0.84	2.04	2.750 (4)	142
O6—H6o⋯O2^ii^	0.82	1.94	2.746 (4)	168
N1—H1n⋯O4	0.88	2.08	2.908 (4)	156
N3—H3n⋯O1	0.88	2.10	2.922 (4)	155

Symmetry codes: (i) 
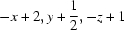
; (ii) 
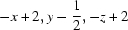
.

**Fig. 1.**
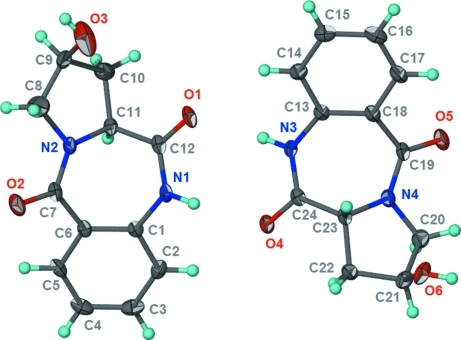
Thermal ellipsoid plot (Barbour, 2001) of the two independent molecules of C12H12N2O3 at the 50% probability level. Hydrogen atoms are drawn as spheres of arbitrary radius.

**Fig. 2.**
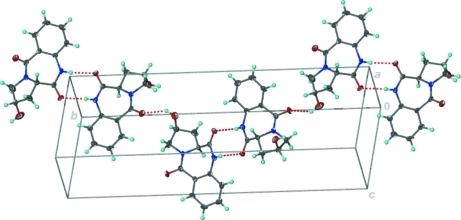
The hydrogen-bonded chain structure.

## supplementary crystallographic information

### Comment

An early study reported the structure of pyrrolo[2,1-*c*][1,4]benzodiazepine-5,11-dione (Feigel *et al.*, 1990). The present structure (Scheme I) is the hydroxy-substituted analog. The seven-membered ring that is fused with the phenylene ring adopts a boat-shaped conformation. The five-membered ring adopts an envelope conformation. The two independent molecules are disposed about a false center-of-inversion and are connected by a pair of N—H···O hydrogen bonds (Fig. 1). Adjacent dimers are linked by a pair of O—H···O hydrogen bonds (Table 1) to generate a chain running along the *b*-axis of the monoclinic unit cell (Fig. 2).

### Experimental

*N*-Carboxyanthranilic anhydride (isatoic anhydride) (3 g,18.4 mmol) and *allo*-4-hydroxy-*L*-proline (2.41 g, 18.4 mmol) were heated in DFM (60 ml). The solvent was evaporated under reduced pressure and the residue was washed with water (60 ml). The product isolated was recrystallized from ethanol to furnish colorless crystals.

### Refinement

Carbon-bound H-atoms were placed in calculated positions (C—H = 0.93–0.98 Å) and were included in the refinement in the riding model approximation, with *U*_iso_(H) set to 1.2*U*_eq_(C). The oxygen- and nitrogen-bound H atoms were similarly treated (N—H = 0.88, O—H = 0.84 Å). Some 1461 Friedel pairs were merged. The absolute configuration was assumed to be that of the *allo*-*L*-proline reactant.

### Crystal data

**Table:**

C_12_H_12_N_2_O_3_	*F*(000) = 488
*M**_r_* = 232.24	*D*_x_ = 1.460 Mg m^−^^3^
Monoclinic, *P*2_1_	Mo *K*α radiation, λ = 0.71073 Å
Hall symbol: P 2yb	Cell parameters from 2147 reflections
*a* = 4.8366 (2) Å	θ = 2.5–27.3°
*b* = 25.7449 (11) Å	µ = 0.11 mm^−^^1^
*c* = 8.5420 (4) Å	*T* = 293 K
β = 96.509 (2)°	Prism, colorless
*V* = 1056.77 (8) Å^3^	0.20 × 0.15 × 0.10 mm
*Z* = 4	

### Data collection

**Table:**

Bruker APEXII CCD-detector diffractometer	2027 reflections with *I* > 2σ(*I*)
Radiation source: fine-focus sealed tube	*R*_int_ = 0.032
graphite	θ_max_ = 27.5°, θ_min_ = 2.4°
φ and ω scans	*h* = −6→6
7493 measured reflections	*k* = −19→33
2435 independent reflections	*l* = −11→9

### Refinement

**Table:**

Refinement on *F*^2^	Primary atom site location: structure-invariant direct methods
Least-squares matrix: full	Secondary atom site location: difference Fourier map
*R*[*F*^2^ > 2σ(*F*^2^)] = 0.044	Hydrogen site location: inferred from neighbouring sites
*wR*(*F*^2^) = 0.110	H-atom parameters constrained
*S* = 1.03	*w* = 1/[σ^2^(*F*_o_^2^) + (0.0581*P*)^2^ + 0.2538*P*] where *P* = (*F*_o_^2^ + 2*F*_c_^2^)/3
2435 reflections	(Δ/σ)_max_ = 0.001
309 parameters	Δρ_max_ = 0.40 e Å^−^^3^
1 restraint	Δρ_min_ = −0.34 e Å^−^^3^

### Fractional atomic coordinates and isotropic or equivalent isotropic displacement parameters (Å^2^)

**Table:**

	*x*	*y*	*z*	*U*_iso_*/*U*_eq_	
O1	0.7223 (5)	0.49980 (10)	0.5932 (3)	0.0327 (6)	
O2	0.4033 (6)	0.65398 (10)	0.9439 (3)	0.0395 (7)	
O3	0.8361 (7)	0.63368 (14)	0.4475 (6)	0.0754 (13)	
H3O	0.8909	0.6615	0.4102	0.113*	
O4	0.6970 (4)	0.39402 (9)	0.8727 (3)	0.0275 (5)	
O5	1.0501 (6)	0.23833 (10)	0.5383 (3)	0.0386 (7)	
O6	1.3550 (5)	0.25061 (10)	1.0454 (3)	0.0363 (6)	
H6O	1.4044	0.2201	1.0495	0.054*	
N1	0.4993 (6)	0.49948 (11)	0.8102 (3)	0.0241 (6)	
H1N	0.6015	0.4725	0.8434	0.029*	
N2	0.4375 (6)	0.60987 (11)	0.7194 (3)	0.0257 (6)	
N3	0.9248 (6)	0.39419 (11)	0.6586 (3)	0.0239 (6)	
H3N	0.8207	0.4208	0.6241	0.029*	
N4	1.0092 (6)	0.28335 (11)	0.7599 (3)	0.0265 (6)	
C1	0.3057 (6)	0.51590 (13)	0.9102 (4)	0.0211 (7)	
C2	0.1926 (7)	0.47711 (14)	0.9988 (4)	0.0281 (8)	
H2	0.2441	0.4427	0.9865	0.034*	
C3	0.0073 (8)	0.48915 (16)	1.1031 (4)	0.0336 (9)	
H3	−0.0655	0.4629	1.1609	0.040*	
C4	−0.0728 (7)	0.54019 (17)	1.1234 (4)	0.0335 (9)	
H4	−0.2035	0.5481	1.1916	0.040*	
C5	0.0436 (7)	0.57897 (14)	1.0412 (4)	0.0280 (8)	
H5	−0.0053	0.6133	1.0573	0.034*	
C6	0.2341 (6)	0.56785 (13)	0.9338 (4)	0.0215 (7)	
C7	0.3665 (7)	0.61335 (14)	0.8647 (4)	0.0266 (8)	
C8	0.5274 (12)	0.65602 (18)	0.6375 (6)	0.0571 (14)	
H8A	0.7034	0.6689	0.6888	0.069*	
H8B	0.3898	0.6834	0.6367	0.069*	
C9	0.5579 (7)	0.63853 (14)	0.4722 (4)	0.0302 (8)	
H9	0.4641	0.6631	0.3962	0.036*	
C10	0.4224 (9)	0.58547 (16)	0.4508 (4)	0.0379 (9)	
H10A	0.5437	0.5618	0.4027	0.045*	
H10B	0.2481	0.5881	0.3829	0.045*	
C11	0.3702 (6)	0.56560 (13)	0.6127 (4)	0.0226 (7)	
H11	0.1733	0.5565	0.6120	0.027*	
C12	0.5502 (6)	0.51928 (13)	0.6704 (4)	0.0229 (7)	
C13	1.1233 (6)	0.37765 (13)	0.5599 (4)	0.0217 (7)	
C14	1.2309 (7)	0.41619 (14)	0.4691 (4)	0.0262 (7)	
H14	1.1749	0.4505	0.4791	0.031*	
C15	1.4186 (7)	0.40417 (16)	0.3649 (4)	0.0324 (9)	
H15	1.4902	0.4303	0.3060	0.039*	
C16	1.5008 (7)	0.35323 (17)	0.3478 (4)	0.0341 (9)	
H16	1.6315	0.3450	0.2798	0.041*	
C17	1.3881 (7)	0.31511 (16)	0.4319 (4)	0.0300 (8)	
H17	1.4390	0.2808	0.4173	0.036*	
C18	1.1984 (7)	0.32618 (13)	0.5392 (4)	0.0247 (7)	
C19	1.0782 (7)	0.27964 (13)	0.6126 (4)	0.0256 (7)	
C20	0.9495 (7)	0.23740 (14)	0.8527 (4)	0.0319 (8)	
H20A	1.0455	0.2070	0.8193	0.038*	
H20B	0.7513	0.2304	0.8439	0.038*	
C21	1.0603 (7)	0.25316 (14)	1.0199 (4)	0.0297 (8)	
H21	0.9728	0.2334	1.0990	0.036*	
C22	0.9835 (7)	0.30997 (14)	1.0207 (4)	0.0257 (7)	
H22A	1.0910	0.3281	1.1067	0.031*	
H22B	0.7870	0.3144	1.0303	0.031*	
C23	1.0551 (6)	0.32964 (12)	0.8612 (4)	0.0224 (7)	
H23	1.2516	0.3398	0.8696	0.027*	
C24	0.8747 (6)	0.37459 (13)	0.7980 (4)	0.0221 (7)	

### Atomic displacement parameters (Å^2^)

**Table:**

	*U*^11^	*U*^22^	*U*^33^	*U*^12^	*U*^13^	*U*^23^
O1	0.0281 (12)	0.0261 (14)	0.0466 (15)	0.0027 (10)	0.0160 (11)	−0.0110 (12)
O2	0.0514 (16)	0.0236 (15)	0.0462 (17)	−0.0087 (12)	0.0166 (13)	−0.0161 (12)
O3	0.053 (2)	0.048 (2)	0.135 (4)	−0.0167 (17)	0.057 (2)	−0.031 (2)
O4	0.0263 (12)	0.0226 (13)	0.0352 (14)	0.0035 (10)	0.0101 (10)	−0.0022 (11)
O5	0.0516 (16)	0.0244 (15)	0.0418 (17)	0.0011 (12)	0.0137 (13)	−0.0114 (12)
O6	0.0276 (12)	0.0255 (14)	0.0564 (17)	0.0064 (10)	0.0072 (11)	0.0143 (13)
N1	0.0227 (13)	0.0198 (15)	0.0295 (15)	0.0064 (11)	0.0014 (11)	−0.0034 (12)
N2	0.0334 (15)	0.0139 (15)	0.0308 (16)	−0.0040 (11)	0.0084 (13)	−0.0038 (12)
N3	0.0249 (13)	0.0200 (15)	0.0269 (14)	0.0083 (11)	0.0030 (11)	0.0014 (12)
N4	0.0299 (15)	0.0195 (16)	0.0313 (15)	0.0002 (11)	0.0090 (12)	−0.0029 (13)
C1	0.0179 (14)	0.0233 (17)	0.0212 (16)	−0.0014 (13)	−0.0012 (12)	−0.0023 (13)
C2	0.0306 (18)	0.0210 (18)	0.0313 (18)	−0.0009 (14)	−0.0031 (15)	0.0015 (15)
C3	0.0305 (18)	0.044 (2)	0.0262 (17)	−0.0118 (16)	0.0016 (15)	0.0053 (16)
C4	0.0247 (17)	0.052 (3)	0.0248 (18)	−0.0047 (17)	0.0069 (14)	−0.0074 (17)
C5	0.0266 (16)	0.031 (2)	0.0259 (18)	0.0038 (14)	0.0028 (14)	−0.0107 (15)
C6	0.0194 (14)	0.0204 (17)	0.0240 (16)	−0.0003 (13)	−0.0004 (12)	−0.0053 (13)
C7	0.0252 (16)	0.0212 (19)	0.0336 (19)	0.0018 (13)	0.0045 (14)	−0.0059 (15)
C8	0.094 (4)	0.029 (3)	0.052 (3)	−0.028 (2)	0.026 (3)	−0.005 (2)
C9	0.0321 (18)	0.0222 (18)	0.038 (2)	0.0071 (14)	0.0096 (15)	0.0070 (15)
C10	0.049 (2)	0.034 (2)	0.032 (2)	−0.0083 (18)	0.0119 (17)	−0.0012 (17)
C11	0.0207 (15)	0.0187 (17)	0.0287 (17)	−0.0016 (12)	0.0037 (12)	−0.0033 (14)
C12	0.0155 (14)	0.0201 (17)	0.0329 (18)	−0.0039 (12)	0.0022 (13)	−0.0083 (14)
C13	0.0186 (15)	0.0252 (18)	0.0207 (16)	0.0009 (13)	0.0001 (12)	−0.0009 (14)
C14	0.0265 (16)	0.0284 (19)	0.0230 (17)	−0.0016 (14)	−0.0009 (13)	0.0010 (14)
C15	0.0309 (18)	0.039 (2)	0.0270 (18)	−0.0058 (16)	0.0024 (15)	0.0028 (16)
C16	0.0264 (17)	0.052 (3)	0.0243 (18)	0.0002 (17)	0.0055 (14)	−0.0045 (17)
C17	0.0283 (17)	0.038 (2)	0.0245 (17)	0.0081 (15)	0.0038 (14)	−0.0052 (15)
C18	0.0236 (16)	0.0271 (19)	0.0235 (17)	0.0034 (13)	0.0028 (13)	−0.0032 (14)
C19	0.0255 (16)	0.0206 (18)	0.0310 (18)	0.0047 (13)	0.0054 (14)	−0.0035 (14)
C20	0.0338 (17)	0.025 (2)	0.038 (2)	−0.0038 (15)	0.0113 (15)	0.0012 (16)
C21	0.0269 (16)	0.0259 (19)	0.038 (2)	−0.0012 (14)	0.0107 (14)	0.0073 (15)
C22	0.0259 (16)	0.028 (2)	0.0230 (17)	0.0043 (14)	0.0034 (13)	−0.0016 (14)
C23	0.0197 (15)	0.0201 (18)	0.0280 (17)	0.0001 (12)	0.0046 (13)	−0.0038 (13)
C24	0.0180 (14)	0.0209 (17)	0.0274 (17)	−0.0046 (12)	0.0018 (13)	−0.0078 (14)

### Geometric parameters (Å, °)

**Table:**

O1—C12	1.226 (4)	C8—C9	1.505 (6)
O2—C7	1.247 (4)	C8—H8A	0.9700
O3—C9	1.391 (4)	C8—H8B	0.9700
O3—H3O	0.8400	C9—C10	1.517 (5)
O4—C24	1.233 (4)	C9—H9	0.9800
O5—C19	1.238 (4)	C10—C11	1.522 (5)
O6—C21	1.419 (4)	C10—H10A	0.9700
O6—H6O	0.8200	C10—H10B	0.9700
N1—C12	1.347 (4)	C11—C12	1.525 (5)
N1—C1	1.402 (4)	C11—H11	0.9800
N1—H1N	0.8800	C13—C18	1.391 (5)
N2—C7	1.328 (4)	C13—C14	1.396 (5)
N2—C8	1.470 (5)	C14—C15	1.377 (5)
N2—C11	1.472 (4)	C14—H14	0.9300
N3—C24	1.340 (4)	C15—C16	1.383 (6)
N3—C13	1.413 (4)	C15—H15	0.9300
N3—H3N	0.8800	C16—C17	1.365 (6)
N4—C19	1.341 (4)	C16—H16	0.9300
N4—C20	1.471 (5)	C17—C18	1.398 (5)
N4—C23	1.475 (4)	C17—H17	0.9300
C1—C2	1.401 (5)	C18—C19	1.500 (5)
C1—C6	1.402 (5)	C20—C21	1.522 (5)
C2—C3	1.369 (5)	C20—H20A	0.9700
C2—H2	0.9300	C20—H20B	0.9700
C3—C4	1.386 (6)	C21—C22	1.509 (5)
C3—H3	0.9300	C21—H21	0.9800
C4—C5	1.377 (5)	C22—C23	1.530 (5)
C4—H4	0.9300	C22—H22A	0.9700
C5—C6	1.401 (4)	C22—H22B	0.9700
C5—H5	0.9300	C23—C24	1.512 (5)
C6—C7	1.489 (5)	C23—H23	0.9800
			
C9—O3—H3O	109.5	C10—C11—C12	114.1 (3)
C21—O6—H6O	109.5	N2—C11—H11	109.6
C12—N1—C1	128.9 (3)	C10—C11—H11	109.6
C12—N1—H1N	115.6	C12—C11—H11	109.6
C1—N1—H1N	115.6	O1—C12—N1	121.7 (3)
C7—N2—C8	120.8 (3)	O1—C12—C11	122.9 (3)
C7—N2—C11	124.6 (3)	N1—C12—C11	115.3 (3)
C8—N2—C11	112.8 (3)	C18—C13—C14	119.2 (3)
C24—N3—C13	128.3 (3)	C18—C13—N3	124.6 (3)
C24—N3—H3N	115.8	C14—C13—N3	116.0 (3)
C13—N3—H3N	115.8	C15—C14—C13	120.9 (3)
C19—N4—C20	122.2 (3)	C15—C14—H14	119.5
C19—N4—C23	124.8 (3)	C13—C14—H14	119.5
C20—N4—C23	111.1 (3)	C14—C15—C16	120.0 (3)
C2—C1—C6	119.0 (3)	C14—C15—H15	120.0
C2—C1—N1	116.3 (3)	C16—C15—H15	120.0
C6—C1—N1	124.6 (3)	C17—C16—C15	119.3 (3)
C3—C2—C1	121.0 (3)	C17—C16—H16	120.3
C3—C2—H2	119.5	C15—C16—H16	120.3
C1—C2—H2	119.5	C16—C17—C18	121.9 (4)
C2—C3—C4	120.6 (4)	C16—C17—H17	119.0
C2—C3—H3	119.7	C18—C17—H17	119.0
C4—C3—H3	119.7	C13—C18—C17	118.6 (3)
C5—C4—C3	119.2 (3)	C13—C18—C19	126.1 (3)
C5—C4—H4	120.4	C17—C18—C19	115.2 (3)
C3—C4—H4	120.4	O5—C19—N4	121.2 (3)
C4—C5—C6	121.5 (3)	O5—C19—C18	119.8 (3)
C4—C5—H5	119.2	N4—C19—C18	119.0 (3)
C6—C5—H5	119.2	N4—C20—C21	102.8 (3)
C5—C6—C1	118.7 (3)	N4—C20—H20A	111.2
C5—C6—C7	116.3 (3)	C21—C20—H20A	111.2
C1—C6—C7	124.7 (3)	N4—C20—H20B	111.2
O2—C7—N2	121.8 (3)	C21—C20—H20B	111.2
O2—C7—C6	119.1 (3)	H20A—C20—H20B	109.1
N2—C7—C6	119.1 (3)	O6—C21—C22	106.7 (3)
N2—C8—C9	105.7 (3)	O6—C21—C20	111.9 (3)
N2—C8—H8A	110.6	C22—C21—C20	101.7 (3)
C9—C8—H8A	110.6	O6—C21—H21	112.0
N2—C8—H8B	110.6	C22—C21—H21	112.0
C9—C8—H8B	110.6	C20—C21—H21	112.0
H8A—C8—H8B	108.7	C21—C22—C23	103.7 (3)
O3—C9—C8	111.6 (4)	C21—C22—H22A	111.0
O3—C9—C10	108.2 (3)	C23—C22—H22A	111.0
C8—C9—C10	107.2 (3)	C21—C22—H22B	111.0
O3—C9—H9	109.9	C23—C22—H22B	111.0
C8—C9—H9	109.9	H22A—C22—H22B	109.0
C10—C9—H9	109.9	N4—C23—C24	111.5 (3)
C9—C10—C11	107.9 (3)	N4—C23—C22	102.8 (3)
C9—C10—H10A	110.1	C24—C23—C22	113.1 (3)
C11—C10—H10A	110.1	N4—C23—H23	109.8
C9—C10—H10B	110.1	C24—C23—H23	109.8
C11—C10—H10B	110.1	C22—C23—H23	109.8
H10A—C10—H10B	108.4	O4—C24—N3	120.9 (3)
N2—C11—C10	104.7 (3)	O4—C24—C23	122.2 (3)
N2—C11—C12	109.1 (2)	N3—C24—C23	116.8 (3)
			
C12—N1—C1—C2	148.1 (3)	C24—N3—C13—C18	36.1 (5)
C12—N1—C1—C6	−36.1 (5)	C24—N3—C13—C14	−149.2 (3)
C6—C1—C2—C3	2.3 (5)	C18—C13—C14—C15	−2.8 (4)
N1—C1—C2—C3	178.3 (3)	N3—C13—C14—C15	−177.8 (3)
C1—C2—C3—C4	0.0 (5)	C13—C14—C15—C16	0.7 (5)
C2—C3—C4—C5	−2.2 (5)	C14—C15—C16—C17	1.8 (5)
C3—C4—C5—C6	2.1 (5)	C15—C16—C17—C18	−2.2 (5)
C4—C5—C6—C1	0.2 (5)	C14—C13—C18—C17	2.4 (4)
C4—C5—C6—C7	−174.0 (3)	N3—C13—C18—C17	176.9 (3)
C2—C1—C6—C5	−2.3 (4)	C14—C13—C18—C19	−172.5 (3)
N1—C1—C6—C5	−178.0 (3)	N3—C13—C18—C19	2.0 (5)
C2—C1—C6—C7	171.3 (3)	C16—C17—C18—C13	0.1 (5)
N1—C1—C6—C7	−4.3 (5)	C16—C17—C18—C19	175.6 (3)
C8—N2—C7—O2	−8.4 (6)	C20—N4—C19—O5	12.2 (5)
C11—N2—C7—O2	−172.4 (3)	C23—N4—C19—O5	175.1 (3)
C8—N2—C7—C6	170.3 (4)	C20—N4—C19—C18	−166.2 (3)
C11—N2—C7—C6	6.4 (5)	C23—N4—C19—C18	−3.2 (5)
C5—C6—C7—O2	30.1 (5)	C13—C18—C19—O5	145.2 (3)
C1—C6—C7—O2	−143.7 (3)	C17—C18—C19—O5	−29.8 (4)
C5—C6—C7—N2	−148.7 (3)	C13—C18—C19—N4	−36.4 (5)
C1—C6—C7—N2	37.5 (5)	C17—C18—C19—N4	148.5 (3)
C7—N2—C8—C9	−173.7 (3)	C19—N4—C20—C21	145.8 (3)
C11—N2—C8—C9	−8.0 (5)	C23—N4—C20—C21	−19.3 (3)
N2—C8—C9—O3	−105.8 (4)	N4—C20—C21—O6	−76.1 (3)
N2—C8—C9—C10	12.6 (5)	N4—C20—C21—C22	37.4 (3)
O3—C9—C10—C11	107.7 (4)	O6—C21—C22—C23	75.0 (3)
C8—C9—C10—C11	−12.9 (5)	C20—C21—C22—C23	−42.3 (3)
C7—N2—C11—C10	165.1 (3)	C19—N4—C23—C24	67.3 (4)
C8—N2—C11—C10	0.1 (4)	C20—N4—C23—C24	−128.1 (3)
C7—N2—C11—C12	−72.3 (4)	C19—N4—C23—C22	−171.3 (3)
C8—N2—C11—C12	122.7 (4)	C20—N4—C23—C22	−6.7 (3)
C9—C10—C11—N2	7.9 (4)	C21—C22—C23—N4	30.4 (3)
C9—C10—C11—C12	−111.4 (3)	C21—C22—C23—C24	150.7 (3)
C1—N1—C12—O1	−177.9 (3)	C13—N3—C24—O4	178.4 (3)
C1—N1—C12—C11	−0.6 (5)	C13—N3—C24—C23	1.2 (5)
N2—C11—C12—O1	−116.6 (3)	N4—C23—C24—O4	117.9 (3)
C10—C11—C12—O1	0.1 (4)	C22—C23—C24—O4	2.7 (4)
N2—C11—C12—N1	66.1 (4)	N4—C23—C24—N3	−64.9 (4)
C10—C11—C12—N1	−177.1 (3)	C22—C23—C24—N3	179.9 (3)

### Hydrogen-bond geometry (Å, °)

**Table:**

*D*—H···*A*	*D*—H	H···*A*	*D*···*A*	*D*—H···*A*
O3—H3o···O5^i^	0.84	2.04	2.750 (4)	142
O6—H6o···O2^ii^	0.82	1.94	2.746 (4)	168
N1—H1n···O4	0.88	2.08	2.908 (4)	156
N3—H3n···O1	0.88	2.10	2.922 (4)	155

Symmetry codes: (i) −*x*+2, *y*+1/2, −*z*+1; (ii) −*x*+2, *y*−1/2, −*z*+2.
